# Cortical Thickness in Alcohol Dependent Patients With Apathy

**DOI:** 10.3389/fpsyt.2020.00364

**Published:** 2020-05-05

**Authors:** Kebing Yang, Qingyan Yang, Yajuan Niu, Fengmei Fan, Song Chen, Xingguang Luo, Shuping Tan, Zhiren Wang, Jinghui Tong, Fude Yang, Thang M. Le, Chiang-Shan R. Li, Yunlong Tan

**Affiliations:** ^1^Peking University Huilongguan Clinical Medical School, Beijing Huilongguan Hospital, Beijing, China; ^2^Department of Psychiatry, Yale University School of Medicine, New Haven, CT, United States; ^3^Department of Neuroscience, Yale University School of Medicine, New Haven, CT, United States

**Keywords:** alcohol dependence, apathy, occipito-temporal cortex, superior parietal cortex, inferior parietal cortex

## Abstract

**Objectives:**

Many studies reported structural brain changes in patients with alcohol dependence (PADs). However, no studies identified structural correlates of apathy that might aggravate alcohol misuse. Here, we explored regional differences in cortical thickness in PADs relative to healthy controls (HCs), and examined the potential correlation of regional thickness with the severity of apathy.

**Methods:**

Magnetic resonance imaging data were collected from 33 male PADs and 35 male age- and education-matched HCs. We used the FreeSurfer software to investigate group differences in cortical thickness across 148 regions. Apathy was evaluated using the Lille Apathy Rating Scale-Informant (LARS-I). Regression analyses examined the relationship between cortical thickness of regions of interest and apathy score in PADs.

**Results:**

Compared to HCs, PADs showed significant decreases in the cortical thickness of occipito-temporal cortex (OTC), including the left middle occipital gyrus and occipital pole, right superior occipital gyri, and bilateral lingual gyrus; bilateral superior parietal cortex (SPC), including the right intraparietal sulcus; and bilateral inferior parietal cortex (IPC). Furthermore, the cortical thickness of all of the three regions was negatively correlated with the apathy total scores. The cortical thickness of the IPC was also negatively correlated with the action initiation subscore of the LARS-I.

**Conclusions:**

The current results suggest the thickness of bilateral parietal and occipital temporal cortices as neural markers of apathy in PADs. These findings add to the literature by identifying the neural bases of a critical clinical feature of individuals with alcoholism.

## Introduction

Alcohol dependence (AD) is a chronic illness characterized by poor treatment outcomes and high relapse rates (1). Individuals with AD frequently demonstrate motivational deficits, exacerbating personal and interpersonal health (2). Brain imaging was widely used to investigate the consequences of alcohol misuse, which included atrophy of the gray and white matters, sulcal widening, and ventricular enlargement. The structural changes resulted from excessive alcohol consumption (3–7). These deficits involved multiple brain networks that support executive control, salience response, and emotion regulation, which may directly or indirectly contribute to cognitive and motivational dysfunction in patients with AD (8, 9).

The main clinical feature of apathy is lack of motivation (10) and reduced initiative, interest, and/or concern (11). It is also proposed that apathy be defined as dysfunction of volitional processes or a reduction in self-generated, voluntary, and purposeful behavior (i.e., goal-directed behavior) (12). Difficulties in goal-directed behavior, as a key feature of apathy, was significantly associated with symptoms of alcohol dependence (13). Almost all patients with severe alcohol dependence show diminished or lack of living practices except for drinking-related events, and executive dysfunction on alcohol abstinence (2, 14, 15). Importantly, patients undergoing treatment for AD often relapse despite better knowledge and their intention to remain abstinent, reflecting impairments in volition and goal-directed behavior (16). It is thus critical to investigate the neural correlates of apathy in AD.

In imaging studies of various brain disorders, apathy has been associated with structural and function deficits of multiple brain regions (17), particularly the dorsal anterior cingulate cortex (dACC) (18). Other studies implicated the fronto-subcortical circuits and lateral parietal cortex (19). Numerous studies have also shown structural and functional deficits of a wide range of cortical and subcortical circuits in individuals with AD (20). However, no studies to our knowledge have specifically examined the cerebral structural bases of apathy in AD.

The current study addressed this gap in research. On the basis of the literature that goal-directed behaviors require top-down attentional, cognitive and affective control as well as execution of complex motor movements (21), we hypothesized that the frontal and parietal cortical thickness would be diminished and may represent a neurobiological marker of apathy in AD.

## Methods

### Participants and Consents

All patients with AD (PADs) were males, of Han Chinese descent, and recruited from Beijing Huilongguan Hospital between 2017 and 2018. Inclusion criteria were as follows: a) age between 18 and 65 years; b) diagnosis of AD in accordance with Diagnostic and Statistical Manual of Mental Disorders (DSM-IV-TR) criteria (22); c) right-handed; d) abstinent for 14 to 28 days after completion of medically assisted withdrawal, with no obvious withdrawal symptoms and a Clinical Institute Withdrawal Assessment for Alcohol (CIWA) (23) score of less than 3. Patients with other axis I mental disorders, including dependence on substances other than alcohol and nicotine, or severe medical conditions (e.g., unstable hypertension, poorly controlled diabetes, myocardial infarction, liver cirrhosis) were excluded. In addition, we recruited 35 male age- and education-matched healthy controls (HCs) from the general medical clinics of the hospital and local community. Prior to study inclusion, all participants provided written informed consent in accordance with the Declaration of Helsinki and a protocol approved by the ethics committee of Beijing Huilongguan Hospital.

### Assessments

All PADs were evaluated with the Alcohol Use Disorder Identification Test (AUDIT) (24), a semi-structured questionnaire to investigate the severity of alcohol consumption (25). Lifetime Drinking History assessment to determine the number of daily drinks (one drink = 10 g of ethanol), age at first drink, and age at onset of alcohol dependence. The extent of apathy was assessed using the Lille Apathy Rating Scale-Informant (LARS-I) (11), which comprised 33 items across nine domains, each corresponding to a specific clinical dimension of apathy. Each sub-scale score was from −4 to +4, with higher positive scores indicating more severe symptoms. In brief, a negative or positive score indicates less or more apathy, and zero represents moderate apathy in everyday productivity and interests domains or non-applicable or non-classifiable reply in other domains. In this study, we focused on the apathy total score (T score) and action initiation (AI) subscore in data analyses.

### MRI Data Acquisition and Analyses

All PADs (after detoxification) and HCs underwent MRI. MRIs were obtained by using a Siemens 3T MRI scanner. Head motion was minimized using foam pads. Structural MR images were acquired for the whole brain with a sagittal 3D-magnetization prepared rapid acquisition gradient echo (MPRAGE) sequence, using the follow parameters: echo time (TE) = 2.98 ms, inversion time (TI) = 900 ms, repetition time (TR) = 2,300 ms, flip angle (FA) = 9°, field-of-view (FOV) = 240 mm×256 mm, matrix size = 256×240, thickness/gap = 1/0 mm.

Measures of cortical thickness (mm) of 148 cortical regions (bilateral; 74 x 2; Desikan-Killiany Atlas), total subcortical thickness, and total gray matter volume (TGV) were obtained using FreeSurfer (26) (http://surfer.nmr.mgh.harvard.edu). TGV was used as a covariate in all analyses to account for differences in head size. We followed the ENIGMA pipeline (http://enigma.ini.usc.edu/) for quality control of the images: All cortical regions with a thickness >1.5 or <1.5 times the interquartile range were identified and visually inspected by overlaying their segmentations on the participants’ anatomical images. Only imaging data for which segmentation was judged to be accurate were subjected to statistical analyses.

### Statistical Analysis

As all participants were male, continuous demographic variables were analyzed using independent samples t-tests and categorical data were analyzed with Chi-square (*χ^2^*) tests to compare PADs and HCs. The level of statistical significance was set at *p* < 0.05 (two-tailed).

Cortical thickness values of the 148 regions were compared between PADs and HCs using univariate linear regression analysis, where the cortical thickness of each region was used as the dependent variable, and group (PADs/HCs), age, smoking status, education, and TGV were entered as independent variables. The threshold for statistical significance was set at *p* < 0.00033784 (i.e., 0.05/148) to correct for multiple comparisons (Bonferroni correction).

We identified 12 cortical regions of interest (ROIs) that differed significantly between PADs and HCs in thickness, and these regions belong to the occipital temporal cortex (OTC) (lateral cortex including the left middle occipital gyrus, bilateral lingual gyrus, left occipital pole and right superior occipital gyrus), superior parietal cortex (SPC) (lateral cortex including the right intraparietal sulcus and bilateral superior parietal lobule), and inferior parietal cortex (IPC) (lateral cortex including the bilateral angular gyrus and supramarginal gyrus) (27). We performed bivariate correlation analyses to explore the relationship between cortical thickness of the OTC, SPC and IPC and apathy total score (T score), and action initiation subscore (AI score) in PADs. Statistical significance was tested at *p* < 0.05/6, two-sided, to correct for multiple comparisons. As many alcohol drinkers are heavy smokers, we performed two sets of regression, one without smoking status as a covariate, so that the results best reflect the typical populations of alcoholic participants, and the other with smoking status as a covariate, so the effects of smoking could be controlled for.

## Results

### Demographic and Clinical Characteristics

There were no significant differences in age (*t*=1.257, *p* = 0.213) or years of education (*t*=−0.883, *p*=0.381) between PADs and HCs. More PADs were smokers than HCs (*χ^2^* = 11.87, *p*=0.001). Smoking status was thus included as a covariate in the following data analyses. Compared to HCs, PADs showed higher LARS total or T score (*t*=5.85, *p* < 0.001) and action initiation or AI subscore (*t*=5.43, *p* < 0.001). The demographic and clinical characteristics of the two groups are presented in **Table 1**.

**Table 1:** Demographic and clinical characteristics in patients with alcohol dependence (PADs) and healthy controls (HCs) (mean ± SD).

	PAD (n = 33)	HCs (n = 35)	*t/χ^2^*-value	*p*-value
Age, years	44.8 ± 9.8	41.6 ± 11.5	1.257	0.213
Education, years	11.8 ± 3.3	12.3 ± 2.4	−0.883	0.381
Smoker/non-smoker ^a^	26/7	13/22	11.87	0.001
Action initiation (AI)	−1.21 ± 0.86	−2.19 ± 0.61	5.43	0.000
Total score of LARS	−5.85 ± 6.87	−14.78 ± 5.70	5.85	0.000
Age at onset of AD, years	32.5 ± 10.1	N/A	N/A	N/A
Age at first drink, years	19.9 ± 5.5	N/A	N/A	N/A
AUDIT total score	21.7 ± 5.2	N/A	N/A	N/A
Mean daily drinks ^b^	19.1 ± 8.7	N/A	N/A	N/A

PADs, patients with alcohol dependence; HCs, healthy controls; LARS, Lille Apathy Rating Scale; AUDIT, Alcohol Use Disorders Identification Test; N/A, not applicable.

a Chi-square (χ^2^) test: smoker = current or previous smoker; non-smoker: never smoker

b One drink contains 10 g of pure alcohol.

### Cortical Thickness

The cortical thickness of the bilateral lingual gyrus in the OTC was significantly higher in PADs as compared with HCs; however, relative to HCs, PADs exhibited significant decreases in overall cortical thickness in the OTC (consisting of the left middle occipital gyrus and occipital pole, right superior occipital gyri, and bilateral lingual gyrus), SPC, as well as IPC, (all *p*’s <0.00033784, **Table 2**) accounting for age, years of education, smoking status, and TGV. The mean cortical thickness and statistics for all 148 regions were shown in **Supplementary Table 1**.

**Table 2:** Brain regions with significant differences in cortical thickness between patients with alcohol dependence (PADs) and healthy controls (HCs).

Cortical thickness of Destrieux Atlas (mm)	PADs (n 33)	HCs (n = 35)	*F*-value	*p*-value
L-Middle occipital gyrus	2.165 ± 0.297	2.613 ± 0.123	29.545	9.768E−07
L-Lingual gyrus	2.118 ± 0.142	1.879 ± 0.131	44.499	7.960E−09
L-Angular gyrus	2.152 ± 0.329	2.705 ± 0.139	26.065	3.378E−06
L-Supramarginal gyrus	2.371 ± 0.271	2.741 ± 0.141	14.574	3.138E−04
L-Superior parietal lobule	1.931 ± 0.301	2.461 ± 0.117	32.666	3.351E−07
L-Occipital pole	1.672 ± 0.219	1.947 ± 0.145	20.949	2.321E−05
R-Superior occipital gyrus	1.881 ± 0.302	2.261 ± 0.145	17.749	8.313E−05
R-Lingual gyrus	2.137 ± 0.129	1.967 ± 0.156	16.656	1.303E−04
R-Angular gyrus	2.167 ± 0.324	2.704 ± 0.131	41.123	2.208E−08
R-Supramarginal gyrus	2.389 ± 0.299	2.741 ± 0.118	16.734	1.262E−04
R-Superior parietal lobule	1.929 ± 0.318	2.445 ± 0.118	38.473	5.044E−08
R-Intraparietal sulcus and transverse parietal sulci	1.892 ± 0.211	2.191 ± .089	20.290	3.004E−05

One-way ANOVA, Bonferroni corrected at p<0.00033784.

**Figure 1:**
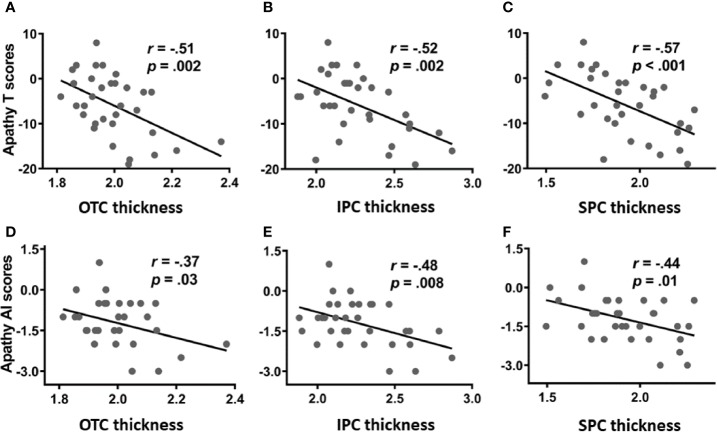
Correlation of cortical thickness of the occipital temporal cortex (OTC), inferior parietal cortex (IPC), and superior parietal cortex (SPC) with apathy total score (T score; upper panel: **A**–**C**) and action initiation subscore (AI subscore; lower panel: **D–F**).

For PADs, we focused on the OTC, SPC, and IPC and performed Pearson correlation analyses to evaluate the relationship between cortical thickness and apathy (T score and AI subscore). Evaluated at *p* < 0.0083 (i.e., 0.05/6, Bonferroni correction), the results revealed negative correlations between the thickness of the OTC, SPC as well as IPC and both apathy T score. The cortical thickness of the IPC was also negatively correlated with the AI subscore. These results are shown in **Figure 1**.

Because many more PADs are smokers, we performed covariance analyses with smoking status as a covariate. The results showed that apathy T score remained negatively correlated with the thickness of the OTC (*r*=−0.44, *p*=0.011), IPC (*r*=−0.47, *p*=0.007), and SPC (*r*=−0.58, *p* < 0.001); and the apathy AI subscore showed negative correlation with thickness of the IPC (*r*=−0.41, *p*=0.019) and SPC (*r*=−0.44, *p*=0.012) but not the OTC (*r*=−0.32, *p*=0.078). The findings suggest that after controlling for smoking status, the thickness of the parietal, but not that of occipital temporal cortex, was correlated with difficulties in action initiation in PADs.

## Discussion

We hypothesized that PADs would exhibit reduced thickness in the frontal and parietal cortex, and the extent of the reduction would be correlated with the severity of apathy in PADs. To this end, we determined differences in regional cortical thickness between PADs and HCs and investigated how these differences are related to apathy in PADs. The results suggest that AD was associated with reduced thickness in the bilateral occipital temporal cortex, the superior and inferior parietal cortices, and the extent of the reduction in cortical thickness was correlated with the severity of apathy. Although the cortical thickness of the bilateral lingual gyrus showed peculiar increases in PADs, this change, contrary to that shown in most of the previous studies (5, 28), did not affect the reduction of the whole OTC region in PADs compared with HCs. The mechanism of compensatory hypertrophy might be a potential explanation to this change (29) because a previous study found similar increases in alcohol abuse individuals, regardless of sex and brain regions (30). Greater reduction of parietal cortical thickness appears to be more specifically related to action initiation deficits in PADs. Together, these findings support structural changes of posterior cortical regions as a neural marker of apathy in alcoholism.

Structural imaging studies reported significant volume losses in the prefrontal cortex, insula, and hippocampus in AD (5, 31, 32); however, volumetric deficits of the posterior cortical regions have received less attention (30). For instance, parietal-occipital gray matter volumes were decreased in patients with AD and can be used to predict shorter time of any alcohol use and relapse to heavy drinking (33). A recent meta-analysis of 3,240 individuals with drug and alcohol dependence indicated that the most severe volumetric deficits are found in not only the frontal but also occipital, temporal, and parietal cortices in those with alcohol dependence (34). Consistently, we observed a significant reduction in cortical thickness in the occipital and parietal cortices. However, it is unclear why structural changes cannot be detected in the frontal cortex. One possibility is that the current work only focused on cortical thickness, a volumetric measure different from that as employed in the earlier work. This discrepancy clearly warrants further studies.

Many studies examined the neural mechanisms underlying cognitive and emotional deficits (35) but few focused on apathy in individuals with AD (36). In accordance with previous studies we observed more significant apathy symptoms, as reflected in the apathy total score in PADs than in HCs, suggesting motivational dysfunction in individuals with alcoholism (37). The cortical thickness of the occipital-temporal as well as superior and inferior parietal cortices was negatively correlated with the severity of apathy, consistent with earlier work associating apathy with structural changes in the parietal cortex, in addition to the prefrontal cortex (PFC) and basal ganglia (38). Research has indicated that the superior parietal lobule, including the intraparietal sulcus, play a key role in goal-directed behaviors and volitional processes (39). In addition, an [18F]-fluorodeoxyglucose (FDG) positron emission tomography (PET) study in a rat model of alcoholism revealed that alcohol significantly reduced whole-brain glucose metabolism, and that the effect was most pronounced in the parietal cortex (40). Together, the current along with these earlier studies support the importance of posterior cortical structures in mediating cognitive and emotional deficits (41), which may manifest as apathy in AD. Importantly, as apathy may impact the motivation for behavioral changes, the current findings are also consistent with our earlier work showing that volumetric deficits in the posterior-occipital region predicted relapse in AD patients undergoing treatment (42).

### Limitations

A few limitations of present study included: 1) there were more smokers in PADs than in HCs. Although we have accounted for the potential effects of smoking in data analyses, one cannot completely rule out the effects of chronic smoking on the findings. On the other hand, compared to smoking, alcohol consumption appears to be an overriding factor leading to alterations in brain structures and functions (43). Further, alcohol dependent individuals are typically smokers; thus, one may argue that the current findings reflect that general alcohol dependent populations; 2) as a specialist hospital in Beijing for the PADs, we were exposed to patients with more serious clinical symptoms and social dysfunction and difficult to enroll those patients who have mild symptoms and no obvious apathy. This is the reason of correlation analysis without setting apathy-free patients as control in this study. 3) because there were much fewer women with alcohol dependence in China and no women were included in the present study, we were unable to examine the effects of gender on cortical thickness in relation to the impact of alcohol misuse. As sex differences in the etiological processes or consequences of alcohol misuse have been demonstrated in numerous studies (44, 45). We hope to study more women to examine sex differences, including the finding that neurotoxicity may be more severe among women with alcoholism. Lastly, the sample size of the present study is moderate and the findings would warrant replication.

## Conclusions

The present study demonstrated significant differences in occipital, temporal, and parietal cortical thickness in individual with alcohol dependence as compared with healthy controls. These structural changes were related to the severity of apathy in alcohol dependent individuals. Future research with functional imaging may provide an opportunity to understand in more details how the posterior cortical regions participate in motivated behavior and how these processes may be compromised in alcohol dependent individuals with apathy.

## Data Availability Statement

All datasets generated for this study are included in the article/**Supplementary Material**.

## Ethics Statement

The studies involving human participants were reviewed and approved by the Ethics Committee of Beijing Huilongguan Hospital. The patients/participants provided their written informed consent to participate in this study. Written informed consent was obtained from the individual(s) for the publication of any potentially identifiable images or data included in this article.

## Author Contributions

YT designed the project, and took responsibility for the integrity of the data and the accuracy of the data analysis. KY, QY, and YN were responsible for recruiting the patients, performing the clinical rating, and collecting the samples. KY wrote the paper. Critical revision of the manuscript for important intellectual content was drafted by YT and C-SL. C-SL, XL, ST, ZW, JT, SC, FF, FY, and TL were invited in evolving the ideas, statistical analysis, and editing the manuscript. All authors have contributed to and have approved the final manuscript.

## Funding

This work was supported by the Beijing Municipal Administration of Hospitals Clinical medicine Development of special funding support (XMLX201834), Beijing Municipal Commission of Science and Technology (Z161100000516046), and the National Institutes of Health (AA021449). None of the funding sources influenced the study design, the collection, analysis, and interpretation of the data, approval of the manuscript, or the decision to submit the manuscript for publication.

## Conflict of Interest

The authors declare that the research was conducted in the absence of any commercial or financial relationships that could be construed as a potential conflict of interest.
