# Supplementary material for: Cortical Thickness in Alcohol Dependent Patients With Apathy
Source: Front Psychiatry. 2020 May 5;11:364. doi: 10.3389/fpsyt.2020.00364 (PMC7214693; doi:10.3389/fpsyt.2020.00364)
Supplement: Supplementary file 1 [file Table_1.doc]

**Supplementary Table 1.** Cortical thickness of brain regions in the PADs and HCs

| Cortical thickness of Desterieux Atlas (mm) | Alcohol dependence (n=33) | Healthy controls (n=35) | | *F*-value | *p*-value |
| --- | --- | --- | --- | --- | --- |
| L-Fronto-marginal gyrus and sulcus | 2.331±0.170 | 2.394±0.130 | | 0.078 | 7.815E-01 |
| L-Inferior occipital gyrus and sulcus | 2.335±0.332 | 2.560±0.184 | | 4.171 | 4.539E-02 |
| L-Paracentral lobule and sulcus | 2.549±0.269 | 2.472±0.164 | | 0.988 | 3.240E-01 |
| L-Subcentral gyrus and sulci | 2.649±0.154 | 2.744±0. 143 | | 0.776 | 3.818E-01 |
| L-Transverse frontopolar gyri and sulci | 2.524±0.229 | 2.660±0.159 | | 0.286 | 5.947E-01 |
| L-Anterior part of the cingulate gyrus and sulcus (ACC) | 2.619±0.159 | 2.752±0.156 | | 2.261 | 1.377E-01 |
| L-Middle-anterior part of the cingulate gyrus and sulcus (aMCC) | 2.539±0.198 | 2.676±0.161 | | 2.700 | 1.054E-01 |
| L-Middle-posterior part of the cingulate gyrus and sulcus (pMCC) | 2.487±0.156 | 2.611±0.124 | | 1.589 | 2.122E-01 |
| L-Posterior-dorsal part of the cingulate gyrus (dPCC) | 2.614±0.390 | 2.947±0.140 | | 5.039 | 2.837E-02 |
| L-Posterior-ventral part of the cingulate gyrus (vPCC) | 2.279±0.605 | 2.645±0.234 | | 0.358 | 5.517E-01 |
| L-Cuneus | 1.990±0.345 | 1.775±0.091 | | 7.850 | 6.773E-03 |
| L-Opercular part of the inferior frontal gyrus | 2.667±0.160 | 2.809±0.148 | | 0.206 | 6.512E-01 |
| L-Orbital part of the inferior frontal gyrus | 2.748±0.170 | 2.834±0.164 | | 0.040 | 8.419E-01 |
| L-Triangular part of the inferior frontal gyrus | 2.568±0.168 | 2.626±0.124 | | 0.971 | 3.282E-01 |
| L-Middle frontal gyrus | 2.473±0.136 | 2.635±0.104 | | 3.357 | 7.174E-02 |
| L-Superior frontal gyrus | 2.775±0.166 | 2.901±0.141 | | 0.872 | 3.540E-01 |
| L-Long insular gyrus and central sulcus of the insula | 3.060±0.267 | 2.978±0.262 | | 1.809 | 1.836E-01 |
| L-Short insular gyri | 3.451±0.179 | 3.388±0.243 | | 2.407 | 1.259E-01 |
| **L-Middle occipital gyrus** | **2.165±0.297** | **2.613±0.123** | | **29.545** | **9.768E-07** |
| L-Superior occipital gyrus | 1.903±0.272 | 2.209±0.161 | | 9.278 | 3.404E-03 |
| L-Lateral occipito-temporal gyrus | 2.822±0.236 | 2.913±0.135 | | 1.094 | 2.997E-01 |
| **L-Lingual gyrus** | **2.118±0.142** | **1.879±0.131** | | **44.499** | **7.960E-09** |
| L-Parahippocampal gyrus | 2.969±0.285 | 3.022±0.192 | | 0.351 | 5.559E-01 |
| L-Orbital gyri | 2.786±0.133 | 2.741±0.153 | | 8.634 | 4.628E-03 |
| **L-Angular gyrus** | **2.152±0.329** | **2.705±0.139** | | **26.065** | **3.378E-06** |
| **L-Supramarginal gyrus** | **2.371±0.271** | **2.741±0.141** | | **14.574** | **3.138E-04** |
| **L-Superior parietal lobule** | **1.931±0.301** | **2.461±0.117** | | **32.666** | **3.351E-07** |
| L-Postcentral gyrus | 2.294±0.344 | 2.263±0.136 | | 0.055 | 8.160E-01 |
| L-Precentral gyrus | 2.689±0.187 | 2.933±0.184 | | 7.930 | 6.512E-03 |
| L-Precuneus | 2.386±0.187 | 2.575±0.129 | | 4.605 | 3.579E-02 |
| L-Straight gyrus | 2.727±0.130 | 2.671±0.120 | | 1.151 | 2.876E-01 |
| L-Subcallosal gyrus | 2.394±0.331 | 2.537±0.306 | | 0.343 | 5.602E-01 |
| L-Anterior transverse temporal gyrus | 2.391±0.284 | 2.495±0.155 | | 0.000 | 9.955E-01 |
| L-Lateral aspect of the superior temporal gyrus | 3.008±0.260 | 3.097±0.178 | | 0.296 | 5.881E-01 |
| L-Planum polare of the superior temporal gyrus | 3.314±0.269 | 3.348±0.226 | | 0.563 | 4.560E-01 |
| L-Planum temporale or temporal plane of the superior temporal gyrus | 2.504±0.193 | 2.609±0.159 | | 0.020 | 8.884E-01 |
| L-Inferior temporal gyrus | 2.888±0.223 | 3.003±0.150 | | 0.000 | 9.891E-01 |
| L-Middle temporal gyrus | 2.889±0.197 | 3.096±0.140 | | 4.086 | 4.756E-02 |
| L-Horizontal ramus of the anterior segment of the lateral sulcus | 2.153±0.194 | 2.235±0.181 | | 0.832 | 3.652E-01 |
| L-Vertical ramus of the anterior segment of the lateral sulcus | 2.228±0.234 | 2.397±0.153 | | 4.283 | 4.268E-02 |
| L-Posterior ramus of the lateral sulcus | 2.370±0.218 | 2.418±0.103 | | 0.056 | 8.143E-01 |
| **L-Occipital pole** | **1.672±0.219** | **1.947±0.145** | | **20.949** | **2.321E-05** |
| L-Temporal pole | 3.288±0.194 | 3.401±.175 | | 0.191 | 6.635E-01 |
| L-Calcarine sulcus | 1.877±0.273 | 1.783±0.132 | | 3.910 | 5.244E-02 |
| L-Central sulcus | 2.226±0.247 | 2.065±.108 | | 6.717 | 1.190E-02 |
| L-Marginal branch of the cingulate sulcus | 2.290±0.218 | 2.321±0.106 | | 0.003 | 9.577E-01 |
| L-Anterior segment of the circular sulcus of the insula | 2.689±0.206 | 2.782±0.190 | | 0.870 | 3.545E-01 |
| L-Inferior segment of the circular sulcus of the insula | 2.724±0.218 | 2.817±.189 | | 0.175 | 6.768E-01 |
| L-Superior segment of the circular sulcus of the insula | 2.542±0.138 | 2.653±0.124 | | 0.636 | 4.284E-01 |
| L-Anterior transverse collateral sulcus | 2.704±0.215 | 2.805±0.161 | | 0.041 | 8.410E-01 |
| L-Posterior transverse collateral sulcus | 2.100±0.357 | 2.111±0.191 | | 7.286 | 8.946E-03 |
| L-Inferior frontal sulcus | 2.181±0.108 | 2.310±0.104 | | 5.614 | 2.094E-02 |
| L-Middle frontal sulcus | 2.124±0.111 | 2.256±0.121 | | 5.459 | 2.271E-02 |
| L-Superior frontal sulcus | 2.335±0.131 | 2.495±0.115 | | 6.922 | 1.073E-02 |
| L-Sulcus intermedius primus | 2.228±0.506 | 2.516±0.353 | | 1.437 | 2.351E-01 |
| L-Intraparietal sulcus and transverse parietal sulci | 1.948±0.238 | 2.228±0.082 | | 6.784 | 1.150E-02 |
| L-Middle occipital sulcus and lunatus sulcus | 1.939±0.332 | 2.093±0.117 | | 1.177 | 2.822E-01 |
| L-Superior occipital sulcus and transverse occipital sulcus | 1.990±0.379 | 2.224±0.117 | | 2.632 | 1.098E-01 |
| L-Anterior occipital sulcus and preoccipital notch | 2.287±0.213 | 2.397±0.139 | | 1.988 | 1.636E-01 |
| L-Lateral occipito-temporal sulcus | 2.525±0.207 | 2.550±0.167 | | 0.673 | 4.153E-01 |
| L-Medial occipito-temporal sulcus and lingual sulcus | 2.317±0.180 | 2.407±0.138 | | 0.008 | 9.298E-01 |
| L-Lateral orbital sulcus | 2.133±0.179 | 2.227±0.218 | | 4.110 | 4.692E-02 |
| L-Medial orbital sulcus | 2.356±0.193 | 2.440±0.133 | | 0.933 | 3.379E-01 |
| L-Orbital sulci | 2.548±0.175 | 2.623±0.161 | | 0.201 | 6.551E-01 |
| L-Parieto-occipital sulcus | 2.207±0.268 | 2.266±0.154 | | 0.006 | 9.397E-01 |
| L-Pericallosal sulcus | 1.960±0.292 | 1.856±0.259 | | 2.059 | 1.564E-01 |
| L-Postcentral sulcus | 2.101±0.130 | 2.208±0.113 | | 3.427 | 6.890E-02 |
| L-Inferior part of the precentral sulcus | 2.328±0.179 | 2.526±0.126 | | 2.571 | 1.139E-01 |
| L-Superior part of the precentral sulcus | 2.412±0.130 | 2.534±0.129 | | 0.737 | 3.940E-01 |
| L-Suborbital sulcus | 2.409±0.261 | 2.440±0.181 | | 0.263 | 6.102E-01 |
| L-Subparietal sulcus | 2.302±0.185 | 2.364±0.121 | | 0.005 | 9.454E-01 |
| L-Inferior temporal sulcus | 2.485±0.158 | 2.570±0.114 | | 0.313 | 5.780E-01 |
| L-Superior temporal sulcus | 2.431±0.134 | 2.549±0.103 | | 0.296 | 5.882E-01 |
| L-Transverse temporal sulcus | 2.273±0.299 | 2.399±0.252 | | 0.013 | 9.092E-01 |
| R-Fronto-marginal gyrus and sulcus | 2.382±0.208 | 2.390±0.151 | | 0.180 | 6.730E-01 |
| R-Inferior occipital gyrus and sulcus | 2.297±0.387 | 2.686±0.189 | | 7.657 | 7.445E-03 |
| R-Paracentral lobule and sulcus | 2.477±0.291 | 2.447±0.170 | | 0.008 | 9.273E-01 |
| R-Subcentral gyrus and sulci | 2.584±0.169 | 2.726±0.148 | | 3.868 | 5.370E-02 |
| R-Transverse frontopolar gyri and sulci | 2.463±0.174 | 2.539±0.122 | | 1.286 | 2.611E-01 |
| R-Anterior part of the cingulate gyrus and sulcus (ACC) | 2.551±0.143 | 2.641±0.125 | | 0.460 | 5.003E-01 |
| R-Middle-anterior part of the cingulate gyrus and sulcus (aMCC) | 2.588±0.143 | 2. 709±0.133 | | 2.644 | 1.090E-01 |
| R-Middle-posterior part of the cingulate gyrus and sulcus (pMCC) | 2.562±0.134 | 2.628±0.127 | | 0.301 | 5.852E-01 |
| R-Posterior-dorsal part of the cingulate gyrus (dPCC) | 2.614±0.307 | 2.881±0.224 | | 1.749 | 1.909E-01 |
| R-Posterior-ventral part of the cingulate gyrus (vPCC) | 2.102±0.810 | 2.679±0.234 | 3.165 | | 8.012E-02 |
| R-Cuneus | 1.987±0.296 | 1.841±0.107 | | 5.118 | 2.719E-02 |
| R-Opercular part of the inferior frontal gyrus | 2.622±0.149 | 2.789±0.135 | | 3.022 | 8.711E-02 |
| R-Orbital part of the inferior frontal gyrus | 2.731±0.223 | 2.768±0.199 | | 1.303 | 2.581E-01 |
| R-Triangular part of the inferior frontal gyrus | 2.543±0.177 | 2.620±0.127 | | 1.030 | 3.140E-01 |
| R-Middle frontal gyrus | 2.494±0.095 | 2.603±0.131 | | 0.138 | 7.112E-01 |
| R-Superior frontal gyrus | 2.762±0.128 | 2.897±0.152 | | 2.832 | 9.743E-02 |
| R-Long insular gyrus and central sulcus of the insula | 3.182±0.306 | 3.141±0.228 | | 1.932 | 1.695E-01 |
| R-Short insular gyri | 3.267±0.287 | 3.243±0.278 | | 3.691 | 5.931E-02 |
| R-Middle occipital gyrus | 2.149±0.408 | 2.647±0.126 | | 13.372 | 5.283E-04 |
| **R-Superior occipital gyrus** | **1.881±0.302** | **2.261±0.145** | | **17.749** | **8.313E-05** |
| R-Lateral occipito-temporal gyrus | 2.789±0.244 | 2.919±0.133 | | 1.311 | 2.567E-01 |
| **R-Lingual gyrus** | **2.137±0.129** | **1.967±0.156** | | **16.656** | **1.303E-04** |
| R-Parahippocampal gyrus | 3.137±0.304 | 3.154±0.181 | | 0.455 | 5.027E-01 |
| R-Orbital gyri | 2.842±0.132 | 2.789±0.146 | | 8.212 | 5.675E-03 |
| **R-Angular gyrus** | **2.167±0.324** | **2.704±0.131** | | **41.123** | **2.208E-08** |
| **R-Supramarginal gyrus** | **2.389±0.299** | **2.741±0.118** | | **16.734** | **1.262E-04** |
| **R-Superior parietal lobule** | **1.929±0.318** | **2.445±0.118** | | **38.473** | **5.044E-08** |
| R-Postcentral gyrus | 2.263±0.455 | 2.211±0.128 | | 0.480 | 4.908E-01 |
| R-Precentral gyrus | 2.653±0.240 | 2.864±0.184 | | 1.368 | 2.467E-01 |
| R-Precuneus | 2.372±0.218 | 2.567±0.107 | | 12.383 | 8.177E-04 |
| R-Straight gyrus | 2.674±0.142 | 2.617±0.182 | | 5.754 | 1.947E-02 |
| R-Subcallosal gyrus | 2.484±0.361 | 2.412±0.457 | | 2.769 | 1.011E-01 |
| R-Anterior transverse temporal gyrus | 2.538±0.299 | 2.590±0.180 | | 0.927 | 3.395E-01 |
| R-Lateral aspect of the superior temporal gyrus | 2.977±0.221 | 3.086±0.169 | | 0.729 | 3.966E-01 |
| R-Planum polare of the superior temporal gyrus | 3.240±0.285 | 3.246±0.175 | | 0.813 | 3.707E-01 |
| R-Planum temporale or temporal plane of the superior temporal gyrus | 2.490±0.180 | 2.599±0.158 | | 0.932 | 3.380E-01 |
| R-Inferior temporal gyrus | 2.788±0.252 | 2.965±0.160 | | 0.388 | 5.357E-01 |
| R-Middle temporal gyrus | 2.812±0.218 | 3.062±0.124 | | 8.317 | 5.393E-03 |
| R-Horizontal ramus of the anterior segment of the lateral sulcus | 2.173±0.193 | 2.324±0.212 | | 3.392 | 7.030E-02 |
| R-Vertical ramus of the anterior segment of the lateral sulcus | 2.330±0.267 | 2.422±0.162 | | 1.075 | 3.038E-01 |
| R-Posterior ramus of the lateral sulcus | 2.468±0.229 | 2.469±0.147 | | 0.482 | 4.902E-01 |
| R-Occipital pole | 1.837±0.207 | 1.965±.104 | | 8.841 | 4.190E-03 |
| R-Temporal pole | 3.355±0.210 | 3.403±0.151 | | 0.031 | 8.609E-01 |
| R-Calcarine sulcus | 1.897±0.288 | 1.819±0.126 | | 4.902 | 3.052E-02 |
| R-Central sulcus | 2.185±0.229 | 2.003±0.133 | | 6.585 | 1.272E-02 |
| R-Marginal branch of the cingulate sulcus | 2.329±0.230 | 2.326±0.102 | | 0.013 | 9.100E-01 |
| R-Anterior segment of the circular sulcus of the insula | 2.735±0.267 | 2.822±0.168 | | 0.017 | 8.980E-01 |
| R-Inferior segment of the circular sulcus of the insula | 2.629±0.285 | 2.706±.213 | | 0.000 | 9.926E-01 |
| R-Superior segment of the circular sulcus of the insula | 2.579±0.141 | 2.700±.112 | | 1.655 | 2.031E-01 |
| R-Anterior transverse collateral sulcus | 2.693±0.232 | 2.729±.188 | | 0.064 | 8.008E-01 |
| R-Posterior transverse collateral sulcus | 2.220±0.287 | 2.180±0.193 | | 3.871 | 5.362E-02 |
| R-Inferior frontal sulcus | 2.145±0.116 | 2.292±.093 | | 14.043 | 3.944E-04 |
| R-Middle frontal sulcus | 2.107±0.102 | 2.183±0.105 | | 3.382 | 7.071E-02 |
| R-Superior frontal sulcus | 2.297±0.134 | 2.427±0.111 | | 0.102 | 7.504E-01 |
| R-Sulcus intermedius primus | 1.977±0.595 | 2.377±0.216 | | 3.340 | 7.244E-02 |
| **R-Intraparietal sulcus and transverse parietal sulci** | **1.892±0.211** | **2.191±.089** | | **20.290** | **3.004E-05** |
| R-Middle occipital sulcus and lunatus sulcus | 1.871±0.390 | 2.166±0.128 | | 4.530 | 3.728E-02 |
| R-Superior occipital sulcus and transverse occipital sulcus | 1.937±0.346 | 2.234±0.124 | | 5.572 | 2.140E-02 |
| R-Anterior occipital sulcus and preoccipital notch | 2.217±0.312 | 2.409±0.150 | | 0.693 | 4.082E-01 |
| R-Lateral occipito-temporal sulcus | 2.515±0.203 | 2.632±0.155 | | 0.414 | 5.222E-01 |
| R-Medial occipito-temporal sulcus and lingual sulcus | 2.327±0.162 | 2.430±0.133 | | 0.072 | 7.892E-01 |
| R-Lateral orbital sulcus | 2.181±0.213 | 2.184±0.213 | | 0.890 | 3.490E-01 |
| R-Medial orbital sulcus | 2.313±0.271 | 2.289±0.157 | | 1.942 | 1.684E-01 |
| R-Orbital sulci | 2.576±0.159 | 2.590±0.101 | | 0.864 | 3.561E-01 |
| R-Parieto-occipital sulcus | 2.198±0.248 | 2.308±0.129 | | 0.798 | 3.750E-01 |
| R-Pericallosal sulcus | 1.901±0.270 | 1.825±0.290 | | 1.494 | 2.263E-01 |
| R-Postcentral sulcus | 2.073±0.230 | 2.119±0.112 | | 0.625 | 4.321E-01 |
| R-Inferior part of the precentral sulcus | 2.281±0.164 | 2.476±0.151 | | 2.598 | 1.121E-01 |
| R-Superior part of the precentral sulcus | 2.344±0.185 | 2.459±0.114 | | 0.024 | 8.786E-01 |
| R-Suborbital sulcus | 2.582±0.470 | 2.549±0.391 | | 0.072 | 7.891E-01 |
| R-Subparietal sulcus | 2.375±0.166 | 2.427±0.120 | | 0.618 | 4.348E-01 |
| R-Inferior temporal sulcus | 2.443±0.152 | 2.584±.120 | | 4.006 | 4.972E-02 |
| R-Superior temporal sulcus | 2.448±0.145 | 2.569±0.110 | | 0.789 | 3.779E-01 |
| R-Transverse temporal sulcus | 2.453±0.280 | 2.567±0.245 | | 0.353 | 5.544E-01 |

Note: Significant *p* values Bonferroni corrected at *p*<0.00033784 are presented in bold.
